# Study on Pyrolysis Characteristics of Phosphate Tailings under H_2_O Atmosphere

**DOI:** 10.3390/ma17092012

**Published:** 2024-04-25

**Authors:** Yanping Yang, Yu Zhang, Dengpan Nie, Chenxin Sun, Jianxin Cao

**Affiliations:** 1 College of Chemistry and Chemical Engineering, Guizhou University, Guiyang 550025, China; m13638129677@163.com (Y.Y.); 13320837671scx@sina.com (C.S.); jxcao@gzu.edu.cn (J.C.); 2 Guizhou Key Laboratory for Green Chemical and Clean Energy Technology, Guiyang 550025, China; 3 College of Chemical Engineering, Guizhou Minzu University, Guiyang 550025, China; ndpz@sina.com; 4 Guizhou Provincial Engineering Research Center of Efficient Utilization for Waster, Guiyang 550025, China

**Keywords:** phosphate tailings, water atmosphere, pyrolysis, physical phase reconstruction and activation

## Abstract

The pyrolysis separation of calcium and magnesium from phosphate tailings is an important process due to its high-value resource utilization. In this paper, aiming to address the problems of high energy consumption, a slow decomposition rate and the low activity of decomposition products in the high-temperature pyrolysis of phosphate tailings, the medium-temperature pyrolysis of phosphate tailings under a H_2_O atmosphere was carried out, and the phase reconstruction and activation of pyrolysis process were discussed. The results showed that compared with N_2_, air and CO_2_ atmospheres, the pyrolysis process of phosphate tailings in a H_2_O atmosphere was changed from two stages to one stage, the starting decomposition temperature was reduced to 500 °C and the decomposition time was shortened to 30 min. The order of the influence of each factor on the pyrolysis of phosphate tailings was temperature > H_2_O pressure > holding time. Under the optimized pyrolysis conditions, the yield of CaMg(CO_3_)_2_ decomposition of phosphate tailings into MgO and CaO was 97.3% and 98.1%, respectively, and the reactivity of MgO was 31.6%. The distribution of Ca and Mg elements in the phosphate tailings after pyrolysis showed a negative correlation, and both of them no longer formed associated compounds; Ca mainly existed in the form of Ca(OH)_2_, Ca_5_(PO_4_)_3_F, CaSiO_3_ and CaF_2_, and Mg mainly existed in the form of MgO, MgF_2_ and Mg(OH)_2_.

## 1. Introduction

The phosphate rock resources in China are abundant but not rich. More than 90.8% of them are medium and low-grade dolomite collophane with a P_2_O_5_ content less than 30% [1,2]. It must be enriched by economic and effective beneficiation to meet the requirements of phosphate ore for wet processing [3]. Phosphate ore flotation will produce a large amount of phosphate tailings. It is reported that for every 1t of phosphate concentrate produced, 0.44t of phosphate tailings will be generated [4]. At present, the utilization of phosphate tailings is mainly focused on pit filling [5], phosphate tailings re-election [6], construction material preparation [7,8], fertilizer production and other uses, with an overall utilization rate of less than 10% [9]. A large number of phosphate tailings are still being processed by stockpiling, resulting in a waste of resources and jeopardizing the safety of the ecological environment [10]. Hence, carrying out basic research to comprehensively utilize and develop phosphate tailings is of great value in accelerating the rational disposal and resource utilization of phosphate tailings, and is also an urgent need to support the sustainable development of the fertilizer industry, chemical industry and even the national economy.

The main mineral composition of phosphate tailings in the Weng’an area of Guizhou Province is dolomite, which accounts for about 80% of the total phosphate tailings [11], and the content of magnesium oxide in the phosphate tailings is 17–20%, which belongs to high-magnesium phosphate tailings [12,13]. The separation and extraction of magnesium from high-magnesium phosphate tailings as a raw material for magnesium series products are a vital way to utilize phosphate tailings with high-value resources. Currently, the methods of magnesium extraction from phosphate tailings are divided into dry and wet methods. The dry method is mainly calcination–carbonization [14], calcination–leaching [15] and the ammonia cycle method [16]; the wet method includes extraction–reverse extraction, nitric acid magnesium removal and so on. The wet process usually uses nitric acid, sulfuric acid and other acidic reagents for the acid leaching of phosphate tailings [17,18,19]; phosphate tailings containing calcium, magnesium, iron and other elements are dissolved at the same time, resulting in subsequent magnesium separation and extraction difficulties, as there will be new solid waste (such as gypsum, etc.) [20], meaning the process is complex. The dry method involves calcining the phosphate tailings at a high temperature first, so that the dolomite in the phosphate tailings decomposes to generate calcium oxide and magnesium oxide, and then separating and extracting calcium and magnesium through digestion, carbonization, ammonium leaching [21], etc. The process route of this method is relatively simple and highly feasible. Nevertheless, the pain point of the dry process is the high energy consumption of the phosphate tailing pyrolysis process as well as the difficulties in the control of the physical phase and physical properties of the product. With a low calcination temperature, the energy provided cannot meet the needs of C-O bond breaking in dolomite; therefore, dolomite decomposition is not complete, and the rate of the pyrolysis reaction is slower [22]. When the temperature is too high, the energy consumption is high, and at the same time, excessive sintering will lead to a decrease in the reactivity of the pyrolysis products [23], affecting the regulation of digestion, carbonization and other subsequent processes.

The pyrolysis atmosphere has a large influence on the decomposition of carbonates. The thermal decomposition of dolomite in air is divided into two stages [24]; the first stage is the decomposition of dolomite into CaCO_3_ and MgO and the release of CO_2_, and the second stage is the further decomposition of CaCO_3_ into CaO and CO_2_. The decomposition temperatures of the two stages are close to each other, with a small difference in temperature, and there are even signs of overlap in the mid-temperature stage. However, under a CO_2_ atmosphere, the decomposition of CaCO_3_ and CaO carbonization in the second stage are reversible reactions [25], while the decomposition of dolomite to CaCO_3_ and MgO in the first stage is an irreversible reaction. Therefore, increasing the partial pressure of CO_2_ can inhibit the decomposition of CaCO_3_ and increase the temperature difference between the two phases of decomposition [26]; so, medium-temperature pyrolysis of dolomite under this atmosphere can initially achieve the phase separation of calcium and magnesium. The results of the previous study of our group show that the calcination of phosphate tailings under a CO_2_ atmosphere to achieve the decomposition of dolomite into CaCO_3_ and MgO is feasible, but the required CO_2_ partial pressure is higher and the reaction time is longer. In addition, later on, when water leaching follows carbonization in the separation process of magnesium and calcium, a part of the CaCO_3_ is prone to enter the liquid phase to form a Ca(HCO_3_)_2_ solution, which leads to the incomplete separation of calcium and magnesium.

It has been shown that water vapor has an accelerating effect on the decomposition of carbonate minerals [27,28,29,30]. Shuai Guo investigated the calcination of limestone in a water vapor atmosphere. The results showed that the presence of water vapor increased the reaction rate and shortened the reaction time, which was caused by the adsorption of H_2_O on the active site of Ca-O* and the formation of hydrogen bonds through the intermolecular interactions of H_2_O [31]. Guido Giammaria studied the effect of water vapor on the decomposition of calcium carbonate. The results showed that the decomposition rate was accelerated fourfold when 1.25% steam concentration was introduced. The apparent activation energy decreased from 201 kJ/mol to 140 kJ/mol, which was caused by the formation of HCO_3_^−^ on the surface of carbonate due to water molecules and the decomposition of HCO_3_^−^ into OH^−^ [32]. Taiga Tone studied the effect of water vapor on the thermal decomposition of five different kinds of calcium carbonate. The results showed that there was an acceleration effect. The mineral aragonite was the most sensitive to water vapor. With the increase in water vapor pressure, the reaction temperature decreased significantly [33]. The generalized kinetic process of the thermal decomposition of calcium carbonate at different temperatures and the values of p(H_2_O) were investigated by introducing the adjusting function (AF) of p(H_2_O), and some meaningful results were obtained. Comparing the above analysis, it was found that the binding force of O* and H_2_O in CO_3_ is stronger than that of CO_2_ [34]. This makes the adsorption rate of dolomite by H_2_O faster and more significant compared to CO_2_ [35]. Therefore, H_2_O can accelerate the release rate of CO_2_ during the thermal decomposition of dolomite by replacing CO_2_.

Based on the above analysis, and in view of the problems of high energy consumption, a slow decomposition rate and the low activity of decomposition products in the high-temperature decomposition of phosphate tailings in the current dry process, the medium-temperature pyrolysis of phosphate tailings under a H_2_O atmosphere was studied in this paper. The influencing factors, phase reconstruction and phase activation of the pyrolysis process were discussed in order to achieve the controllable decomposition and activity regulation of the pyrolysis process of phosphate tailings, and to provide support for the subsequent separation and high-value utilization of calcium and magnesium in phosphate tailings.

## 2. Experimental Materials and Methods

### 2.1. Materials

**Figure 1:**
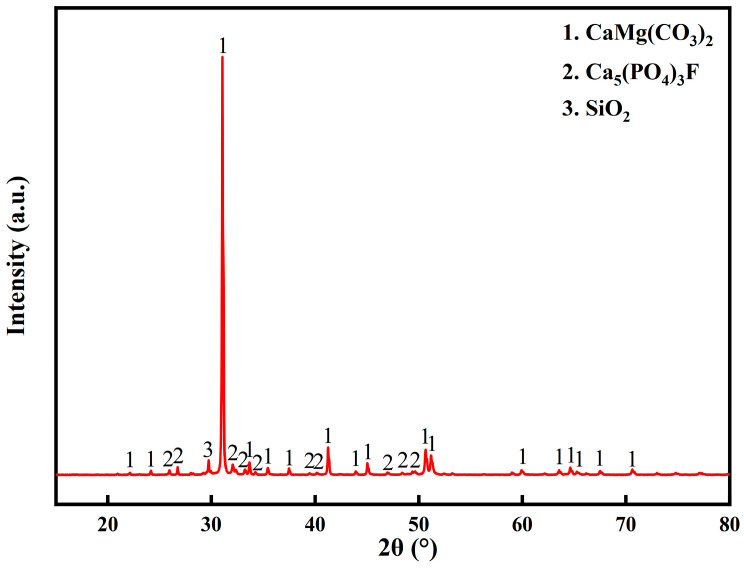
XRD pattern of the phosphate tailings.

Phosphate tailings were taken from Guizhou Chuanheng Co. (Fuquan, China) The main chemical composition and physical phase analysis of the raw materials are shown in Table 1 and Figure 1, respectively. The raw materials were dried at 80 °C for 24 h, mixed and prepared for use after grinding to a particle size of less than 300 mesh.

### 2.2. Experimental Procedure

**Figure 2:**
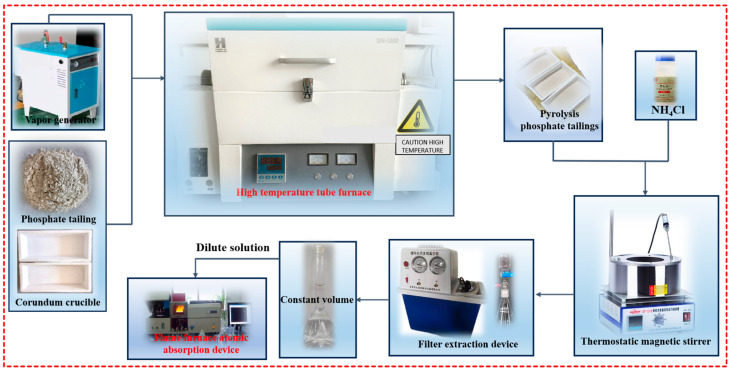
Process flow chart of phosphate tailing pyrolysis experiment.

Figure 2 shows the process flow diagram of the phosphate tailing pyrolysis experiment. The phosphate tailings were pyrolyzed in a high-temperature tubular furnace using a corundum crucible device. The pyrolysis conditions were as follows: steam pressure 0.00–0.04 MPa, heating rate 5–20 K/min, pyrolysis temperature 600–800 °C and holding time 30–120 min. The pyrolyzed phosphate tailings were cooled to room temperature to test their phase composition and phase activity.

### 2.3. Determination of Properties of Pyrolysis Phosphate Tailings

#### 2.3.1. Decomposition Rate of CaMg(CO_3_)_2_ in Phosphate Tailings

The pyrolyzed phosphate tailings treated under different conditions were reacted with ammonium chloride, then filtered and washed. The concentration of Ca^2+^ and Mg^2+^ in the filtrate was detected by flame furnace atomic absorption spectrometry. The measured concentration of Ca^2+^ and Mg^2+^ was converted into the mass of CaO and MgO. Based on the mass of CaO and MgO in the pyrolyzed phosphate tailings treated at 1200 °C, the yield of CaMg(CO_3_)_2_ decomposed into CaO and MgO in phosphate tailings under different experimental conditions was calculated according to Formulas (1) and (2):

(1) $$\omega (CaO)=\frac{m_{CaO}}{m_{1200CaO}}$$

(2) $$\omega (MgO)=\frac{m_{MgO}}{m_{1200MgO}}$$

where ω(CaO) and ω(MgO) represent the yield of CaMg(CO_3_)_2_ decomposition to CaO and MgO, %; m_CaO_ and m_MgO_ are the mass of CaO and MgO leached from the pyrolyzed phosphate tailings under experimental conditions, g; m_1200CaO_ and m_1200MgO_ are the mass of CaO and MgO leached from the pyrolyzed phosphate tailings at 1200 °C, g.

#### 2.3.2. Detection of MgO Activity in Pyrolyzed Phosphate Tailings

According to the standard YB/T 4019-2006 [36], 2.00 g of pyrolyzed phosphate tailings was weighed in a glass weighing bottle, 20 mL of deionized water was added, the lid was covered and a slight gap was left. Hydration was carried out at constant temperature and humidity (temperature 20 °C ± 2 °C; relative humidity 75 ± 5%) for 24 h. The weighing bottle containing the sample was placed in a drying oven, and the sample was dried until it was nearly dry at (105 ± 5) °C, and then the temperature was raised to (150 ± 5) °C until it was completely dry (to a constant amount). The quantity of the samples before and after the hydration test was weighed. The calculation of MgO activity was performed by using Equation (3):

(3) $$\frac{\omega_{2}-\omega_{1}}{0.45\omega_{1}}\times 100$$

In Equation (3), ω_1_ is the mass of the test material, g; ω_2_ is the mass of the test material after hydration, g; 0.45 is the coefficient of the increase in the mass of activated magnesium oxide converted to magnesium oxide after hydration.

### 2.4. Sample Characterization Methods

The pyrolysis characteristics of the phosphate tailings were analyzed using the thermal analyzer NETZSCH STA 449F3 from NETZSCH, Selb, Germany. The test conditions were as follows: phosphate tailings sample mass of 33.50 mg, a temperature range of room temperature—900 °C, a heating rate of 10 K/min, the carrier gas was nitrogen and water vapor, a water vapor flow rate of 7.5 g/h and a N_2_ flow rate of 170 mL/min.

The products were detected by an X-ray diffractometer with the following conditions: a Cu-Kα target as the ray source, a tube current and tube voltage of 40 mA and 40 KV, respectively, a swept range of 5–90° and a scanning speed of 2 °•min^−1^. Finally, the data were analyzed by MDI jade 6 software. Quantitative physical phase analysis of the pyrolytic phosphate tailings was carried out using Rietveld analysis and TOPAS academic software (http://www.topas-academic.net/).

The samples were detected using an X-ray photoelectron spectrometer. The testing conditions were as follows: a power of 200 W, combined with the ability to use C1s (284.8 eV) correction. The results were analyzed using Thermo Avantage v5.9921 software and plotted using the relevant software.

A scanning electron microscope and energy spectrometer (SEM-EDX) were used to analyze the micromorphology and elemental distribution of the samples, and the test conditions were as follows: an acceleration voltage of 0.1–30 kV, an electron beam current of 1–2 nA, a vacuum of the sample of 10^−4^ Pa, an effective area of the energy spectrometry detector of 50 mm^2^, and an energy resolution of Mn Kα better than 127 eV.

The elemental correlation and elemental distribution of the pyrolysis phosphate tailings were analyzed by an electron probe microanalyzer (EPMA) under the following conditions: a voltage of 15 kV, a current of 30 nA and a beam spot diameter of 5 μm.

## 3. Results and Discussion

### 3.1. Characterization of Phosphate Tailing Pyrolysis under H_2_O Atmosphere

#### 3.1.1. Thermodynamic Calculations of Pyrolysis Processes

**Figure 3:**
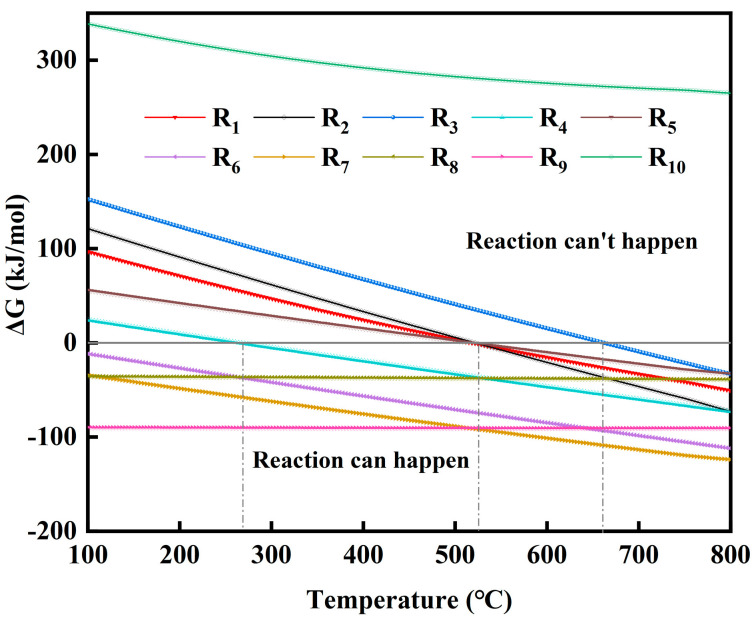
Gibbs free energy versus temperature for each reaction during pyrolysis of phosphate tailings.

The pyrolysis process of phosphate tailings under a H_2_O atmosphere includes the pyrolytic separation of dolomite in phosphate tailings, the pyrolytic activation of the vein minerals (the main chemical composition of SiO_2_) and the interactions between pyrolysis products; the possible reaction equations in the system are shown in Table 2. The thermodynamic calculation of the series of equations was carried out using HSC 6.0 software, and the results are shown in Figure 3.

As can be seen from Figure 3, R_1_ and R_2_ can be spontaneous when the temperature is higher than 518 °C, and R_3_ can be spontaneous when the temperature is higher than 665 °C, indicating that the pyrolysis process of phosphate tailings in a H_2_O atmosphere is mainly the decomposition of CaMg(CO_3_)_2_ under the action of water molecules releasing CO_2_ to generate Ca(OH)_2_ and Mg(OH)_2_. When the temperature is higher than 270 °C and 520 °C, R_4_ and R_5_ can be carried out spontaneously, and the Δ G of the reactions decreases with the increase in temperature, which indicates that increasing the temperature is conducive to the strengthening of the intermediate products Ca(OH)_2_ and Mg(OH)_2_. R_5_ needs to take place at higher temperatures, which is favorable to the preservation of the Ca(OH)_2_ phase in the products. The trends of Δ G values for reactions R_6_, R_7_, R_8_ and R_9_ show that Ca(OH)_2_, Mg(OH)_2_, CaO and MgO will react with SiO_2_ to form the corresponding silicates, with the most pronounced trend in the formation of calcium silicate. R_10_ shows that none of the Ca_5_(PO_4_)_3_F reacts with water throughout the entire temperature range.

**Figure 4:**
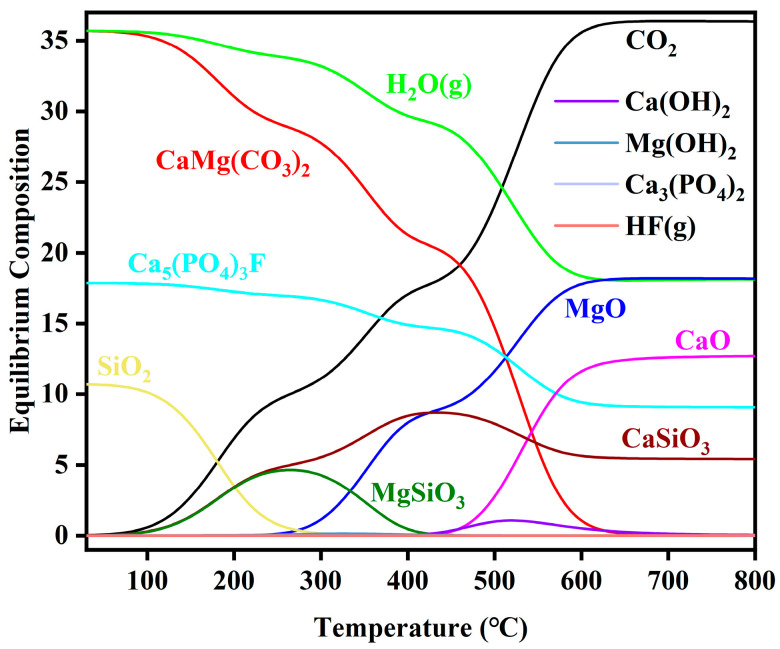
Variation in thermodynamic equilibrium composition of phosphate tailings decomposition with temperature.

The thermodynamic equilibrium composition of phosphate tailings decomposition versus temperature is shown in Figure 4 (calculated material ratios: 1 kmolCaMg(CO_3_)_2_, 1 kmolH_2_O, 0.5 kmolCa_5_(PO_4_)_3_F and 0.3 kmolSiO_2_). The decomposition process of phosphate tailings begins with the reaction of CaMg(CO_3_)_2_ with H_2_O(g) to form Ca(OH)_2_ and Mg(OH)_2_. Mg(OH)_2_ and Ca(OH)_2_ are unstable and have a strong tendency to decompose to form MgO and CaO. In this process, Mg(OH)_2_ decomposes to MgO and H_2_O at a faster rate, and Ca(OH)_2_ is relatively stable and decomposes only at higher temperatures. In addition, SiO_2_ and the intermediate products Ca(OH)_2_ and Mg(OH)_2_ can also react to produce the corresponding silicates. With the increase in temperature, the silicate composition in the system increases first and then decreases. At the same time, with the increase in temperature, MgSiO_3_ is unstable and decomposes into MgO and SiO_2_, while CaSiO_3_ is relatively stable and can be well retained in the system, which is consistent with the subsequent experimental results.

From Figure 4, it can be inferred that the main reactions occurring in the pyrolysis of phosphate tailings under a H_2_O atmosphere are as follows:

CaMg(CO_3_)_2_ + H_2_O → Mg(OH)_2_•Ca(OH)_2_ + CO_2_ (4)

Mg(OH)_2_•Ca(OH)_2_ → Mg(OH)_2_ + Ca(OH)_2_ + CO_2_ (5)

Mg(OH)_2_ → MgO +H_2_O(g) (6)

Ca(OH)_2_ → CaO +H_2_O(g) (7)

In the meantime, there are ongoing intermediate reactions:

SiO_2_ + Ca(OH)_2_ → CaSiO_3_ + H_2_O (8)

SiO_2_ + Mg(OH)_2_ → MgSiO_3_ + H_2_O (9)

SiO_2_ + CaO → CaSiO_3_ + H_2_O (10)

SiO_2_ + MgO → MgSiO_3_ + H_2_O (11)

#### 3.1.2. TG-DTG Analysis of Phosphate Tailings

**Figure 5:**
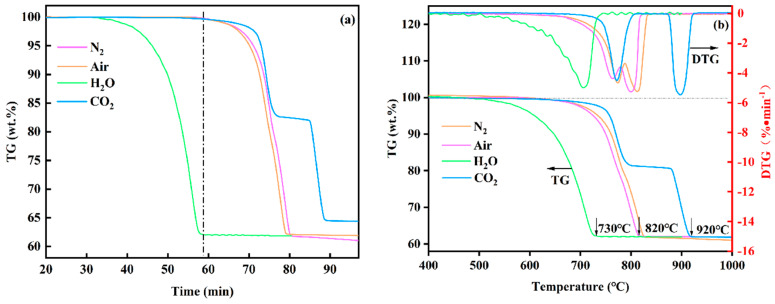
TG (**a**) and TG-DTG (**b**) curves of phosphate tailings under different gas atmospheres.

The TG/DTG curves of phosphate tailings in different atmospheres are shown in Figure 5. As seen in Figure 5a, the thermal decomposition of phosphate tailings in N_2_, air and CO_2_ atmospheres showed a similar trend, with decomposition reactions all starting after 65 min. However, in the H_2_O atmosphere, there was an obvious weight loss in the sample after 30 min, and the mass tended to be constant after the reaction time exceeded 60 min. As seen in Figure 5b, the decomposition of phosphate tailings under the H_2_O atmosphere started at about 500 °C, and the weight loss reached the maximum at 730 °C; meanwhile, in N_2_, air or CO_2_ atmospheres, the decomposition of the samples started after 730 °C, and the main weight loss peaks were all above 800 °C.

The DTG curves also showed that under N_2_, air or CO_2_ atmospheres, the decomposition of phosphate tailings all showed two obvious stages (with two weight loss peaks), and the first stage was mainly the decomposition of CaMg(CO_3_)_2_ in phosphate tailings, releasing CO_2_ to generate MgO and CaCO_3_. The second stage was the decomposition of CaCO_3_. However, in the H_2_O atmosphere, the decomposition of phosphate tailings had only one stage (only one weight loss peak). Combined with the results of the thermodynamic analysis, it can be concluded that this stage was mainly due to the release of CO_2_ from CaMg(CO_3_)_2_ in phosphate tailings under the action of water molecules to generate Ca(OH)_2_ and MgO, as well as the further decomposition of the intermediate product Ca(OH)_2_. The above analysis showed that compared with other atmospheres, the pyrolysis of phosphate tailings in the H_2_O atmosphere was faster, the pyrolysis temperature was lower and the complete pyrolysis time was shorter, which indicates that water vapor had a promoting effect on the pyrolysis of phosphate tailings.

### 3.2. Effect of Pyrolysis Conditions on Phase Reconstruction and Activity of Phosphate Tailings

#### 3.2.1. Effect of H_2_O Pressure

**Figure 6:**
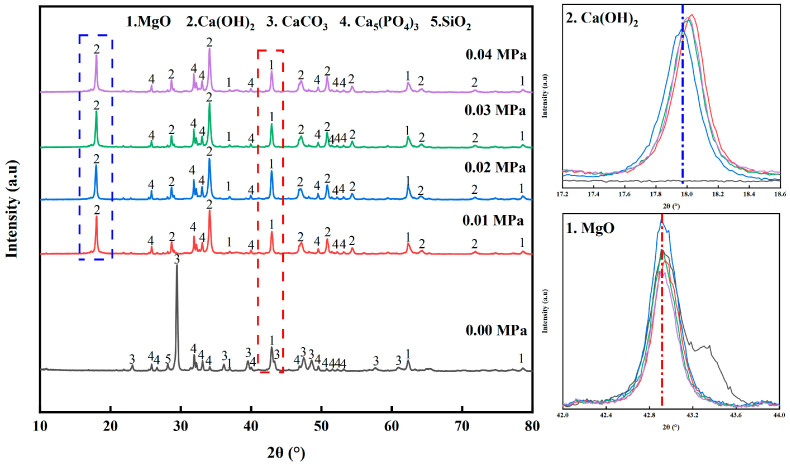
XRD patterns of pyrolyzed phosphate tailings at different H_2_O pressures.

Figure 6 shows the XRD diagrams of the phosphate tailings after pyrolysis under different H_2_O pressures (heating rate of 10 K/min, pyrolysis temperature of 750 °C and holding time of 60 min), from which it can be seen that the mineral composition of phosphate tailings after pyrolysis under a H_2_O atmosphere underwent a large change. Without H_2_O, the main phase composition of the sample was MgO and CaCO_3_, in addition to Ca_5_(PO_4_)_3_F and SiO_2_; with the H_2_O atmosphere, the pyrolysis products of the phosphate tailings were mainly MgO and Ca(OH)_2_, which is attributed to the fact that CaCO_3_ was produced through the pyrolysis of phosphate tailings combined with H_2_O to generate Ca(OH)_2_ and promote the release of CO_2_. Ca(OH)_2_ was further decomposed to produce CaO and H_2_O, and at the same time, the highly active CaO and H_2_O had a tendency to combine to produce Ca(OH)_2_ in the reverse direction.

As can be seen in Figure 6, the peaks of Ca(OH)_2_ and MgO showed a tendency to enhance and then weaken with the increase in H_2_O pressure. This is explained by the continuous passage of H_2_O into the system, which adsorbs and dissociates into OH^−^ and H^+^ on the active sites of CaMg(CO_3_)_2_. H^+^ has a small radius and diffuses more readily to the surface of calcium and magnesium carbonates to form HCO_3_^−^ [37], resulting in the CO_2_ therein escaping more readily to the outside [38]. OH^−^ from the dissociation of H_2_O recombines with Ca^2+^ and Mg^2+^ to form Ca(OH)_2_ and Mg(OH)_2_, but Mg(OH)_2_ is extremely unstable and will continue to decompose into MgO. In addition, the peak of Ca_5_(PO_4_)_3_F in the graph remains almost unchanged, which is due to the fact that the pyrolysis conditions at this point in time do not reach the conditions required for its decomposition. It is noteworthy that the peak of SiO_2_ is significantly weakened, which may be caused by the reaction of SiO_2_ with the products Ca(OH)_2_ or CaO to form CaSiO_3_.

**Figure 7:**
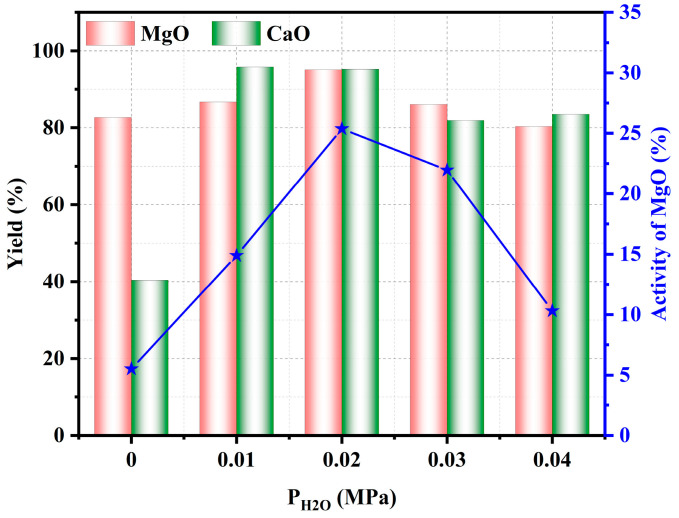
Influence of H_2_O pressure on pyrolysis properties of phosphate tailings.

Figure 7 shows the effect of H_2_O pressure on the yield of MgO and CaO and the activity of MgO. The yield of MgO in the pyrolyzed phosphate tailings increased from 82% to 95.7% when the H_2_O pressure was increased from 0.00 MPa to 0.02 MPa; and the yield of CaO increased from 39% to 95.12%. Both MgO and CaO yields in the pyrolyzed phosphate tailings showed a decreasing trend after the H_2_O pressure exceeded 0.02 MPa. This is attributed to the fact that the H_2_O pressure of 0.02 MPa had provided enough OH^−^ to accommodate the decomposition of CaMg(CO_3_)_2_ to form Ca(OH)_2_ and MgO. Nevertheless, too high a H_2_O pressure may promote the reaction of MgO and CaO with SiO_2_ to form MgSiO_3_ and CaSiO_3_ in the system, leading to a decrease in the yield of MgO and CaO. In addition, the activity of MgO in the pyrolyzed phosphate tailings reached the maximum value of 25.5% when the H_2_O pressure was 0.02 MPa. Continuing to increase the H_2_O pressure, the activity of MgO decreased to 10.3%. Taking all of these considerations into account, the H_2_O pressure should be controlled at around 0.02 MPa.

#### 3.2.2. Effect of Pyrolysis Temperature

**Figure 8:**
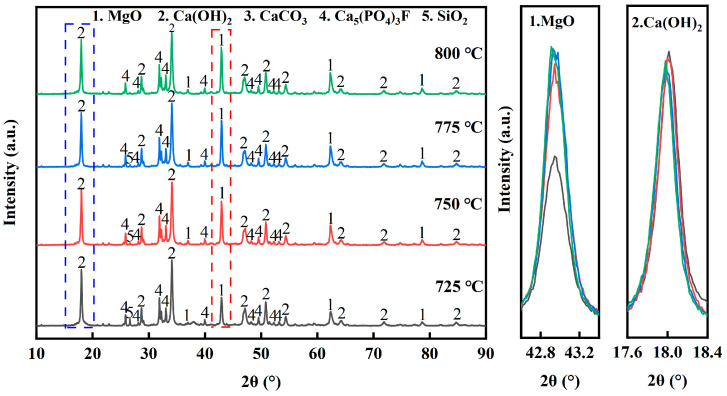
XRD patterns of pyrolyzed phosphate tailings at different temperatures.

Figure 8 shows the XRD diagrams of the pyrolyzed phosphate tailings at different pyrolysis temperatures (0.02 MPaH_2_O, heating rate 10 K/min and holding time 60 min). As can be seen from Figure 8, the main physical phase components were MgO, Ca(OH)_2_, Ca_5_(PO_4_)_3_F and SiO_2_. When the temperature was increased from 725 °C to 800 °C, it can be seen from the local magnification diagram that the peaks of MgO were gradually strengthened, while the peaks of Ca(OH)_2_ were weakened. In addition to that, the peaks of SiO_2_ showed a tendency to weaken or even disappear. This may be due to the fact that the increase in temperature promotes the crystallization of MgO, while Ca(OH)_2_ and SiO_2_ have an enhanced tendency to react with the increase in temperature to form CaSiO_3_, which leads to the enhancement of the characteristic peaks of MgO and the weakening of the characteristic peaks of Ca(OH)_2_ and SiO_2_.

**Figure 9:**
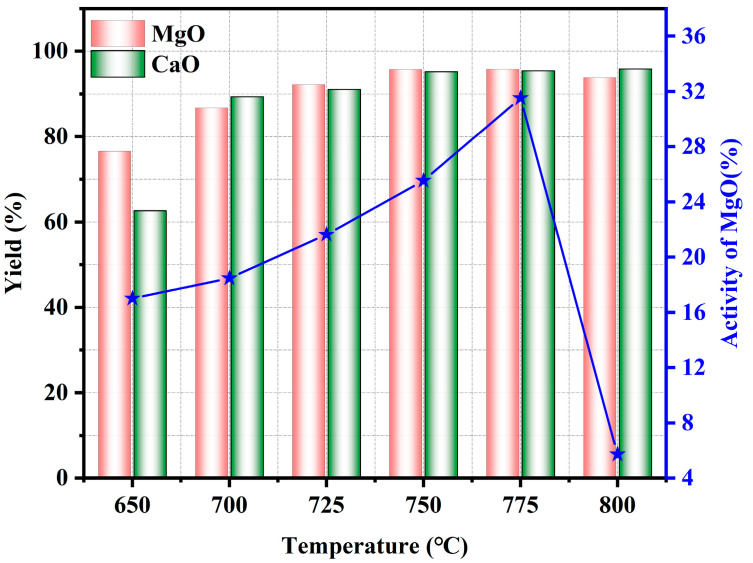
Influence of pyrolysis temperature on pyrolysis properties of phosphate tailings.

Figure 9 shows the effect of pyrolysis temperature on the pyrolysis properties of phosphate tailings. With the increase in pyrolysis temperature, the yields of CaO and MgO showed a tendency to increase first and then to equilibrate. When the temperature was increased from 650 °C to 700 °C, the yield of CaO in the pyrolyzed phosphate tailings increased from 62% to 89%, and the yield of MgO increased from 76% to 87%. Continuing to increase the temperature, the yield of both increased slowly. This is because as the temperature rose, CaMg(CO_3_)_2_ in the phosphate tailings was gradually decomposed into MgO and Ca(OH)_2_, and Ca(OH)_2_ was further decomposed into CaO. It can also be found from Figure 9 that the activity of MgO showed a trend of gradual growth followed by a sharp drop with the increase in temperature. It reached a maximum value of 31.5% at a temperature of 775 °C, and the MgO activity declined to 5.7% above this temperature. Therefore, in order to maximize the yield of CaO and MgO as well as the activity of MgO in the pyrolyzed phosphate tailings, the pyrolysis temperature should be controlled at about 775 °C.

#### 3.2.3. Effect of Holding Time

**Figure 10:**
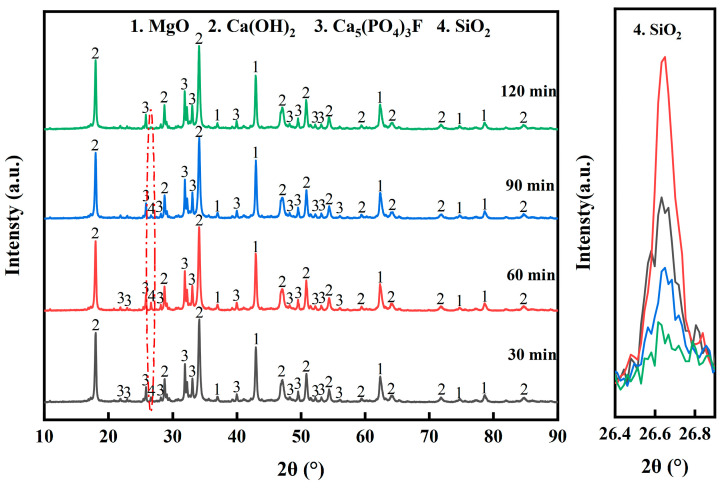
XRD patterns of pyrolyzed phosphate tailings at different holding times.

Figure 10 shows the XRD patterns of the pyrolyzed phosphate tailings at different holding times (0.02 MPa H_2_O, heating rate of 10 K/min and pyrolysis temperature of 775 °C). When the holding time was increased from 30 min to 60 min, the peaks of MgO and Ca(OH)_2_ in the product were slightly enhanced, and the peak of SiO_2_ was more obvious. Continuing to extend the holding time, the peaks of all three showed a tendency to weaken. Especially when the holding time was more than 60 min, the peak of SiO_2_ was obviously weakened, which further indicated that SiO_2_ could react with Ca(OH)_2_ to form CaSiO_3_.

**Figure 11:**
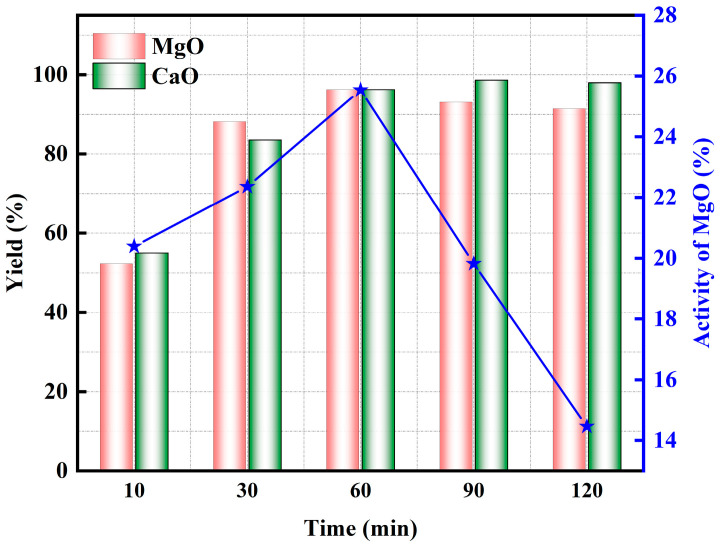
Influence of holding time on pyrolysis properties of phosphate tailings.

Figure 11 shows the effect of holding time on the pyrolysis properties of phosphate tailings. When the holding time was extended from 10 min to 60 min, the yield of MgO and CaO in the pyrolyzed phosphate tailings increased from 52.31% and 54.99% to 96.24%, respectively, and the activity of MgO increased from 20.39% to 25.54%. This result indicates that an appropriate prolongation of the holding time is favorable to promote the decomposition of the reactants [39]. When the holding time exceeded 60 min, the yield of MgO and CaO decreased, and the activity of MgO reduced linearly, indicating that too long a holding time is not conducive to the maintenance of the activity of MgO. Therefore, the optimal holding time is 60 min.

#### 3.2.4. Effect of Heating Rate

**Figure 12:**
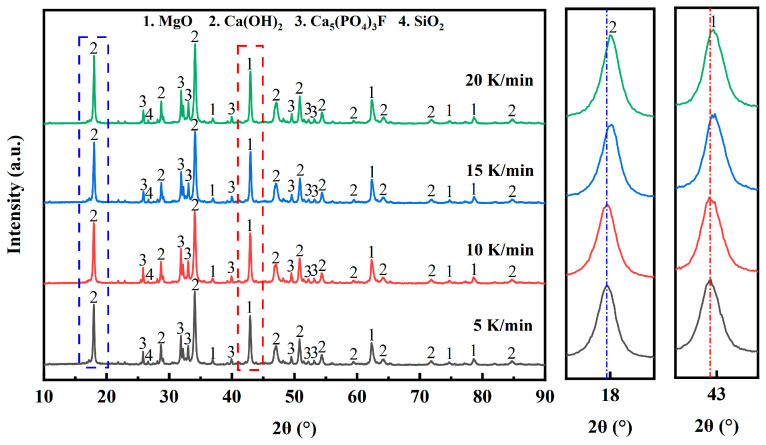
XRD patterns of pyrolyzed phosphate tailings at different heating rates.

Figure 12 shows the XRD patterns of the pyrolyzed phosphate tailings at different heating rates (0.02 MPa H_2_O, pyrolysis temperature 750 °C and holding time 60 min). As can be seen from Figure 12, the heating rate has a certain effect on the crystal surface position of MgO and Ca(OH)_2_, and their characteristic peaks can be gradually observed to shift to the right as a whole when increasing the heating rate from 5 K/min to 20 K/min. This may be attributed to the fact that too high a heating rate makes the substances unevenly heated and insufficiently decomposed. The effect of water vapor makes the surface plasmonic arrangement of the pyrolysis products’ (Ca(OH)_2_ and MgO) particles disordered, and the increase in surface energy makes the product ions adsorb OH^−^ on the surface to form lattice defects such as positive ion vacancies or grain boundary defects, which leads to the rightward shift of the characteristic peaks [40].

**Figure 13:**
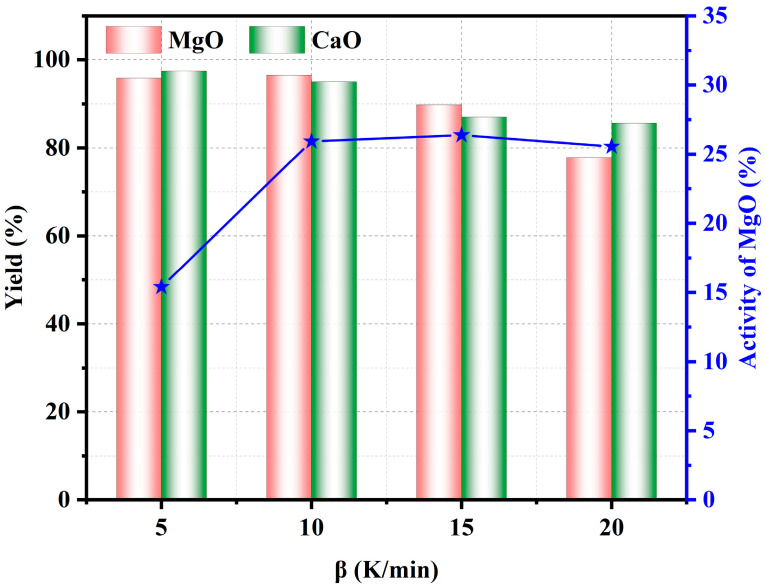
Influence of heating rate on pyrolysis properties of phosphate tailings.

Figure 13 shows the effect of heating rate on the pyrolysis properties of phosphate tailings. As can be seen from Figure 13, the yield of CaO and MgO in the pyrolyzed phosphate tailings decreased with the increase in the heating rate, while the activity of MgO showed the opposite trend. When the heating rate was 5 K/min, the yield of CaO and MgO was relatively high, but the activity of MgO was the lowest. When the heating rate was increased to 10 K/min, the yield of CaO and MgO decreased slightly, but both exceeded 95%, and the activity of MgO increased significantly. These results indicate that at a lower heating rate, the temperature gradient within each substance in the phosphate tailings was very small, and although the reactants decomposed sufficiently at this time, the lattice defects of the products were reduced due to their well-developed lattice, which made their reactivity lower. The activity of MgO remained almost unchanged when the heating rate exceeded 10 K/min, while the yield of CaO and MgO decreased significantly. Therefore, a heating rate of 10 K/min is a suitable choice.

### 3.3. Response Surface Method Optimization and Analysis of Phosphate Tailing Pyrolysis Process Conditions under H_2_O Atmosphere

On the basis of a single-factor experimental analysis, seventeen groups of experiments with three levels of three influencing factors, namely, pyrolysis temperature, holding time and H_2_O pressure, were determined, and the yield of MgO was taken as the response value, and Box–Behnken response surface design and data analysis were applied in Design Expert 13.0 software. The specific experimental factors and the values of each level are shown in Table 3, and the experimental design scheme and experimental results are shown in Table 4.

**Figure 14:**
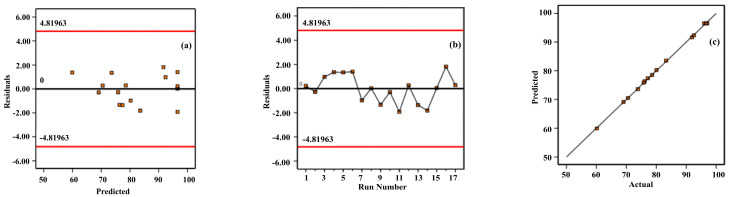
Diagnostic modeling of MgO yield in pyrolysis phosphate tailings. (**a**) Residuals and predicted values; (**b**) residuals and number of experiments; (**c**) actual and predicted values.

The model was analyzed by using Design Expert 13.0 software and the analytical optimization results are displayed in Figure 14 and Table 5. From the distribution of residuals versus predicted values in Figure 14a, the residual values were uniformly distributed at both ends of the 0 scale, and there was no anomaly in the residuals, which indicates that the fit is good and the residual values are reasonable. From the relationship between the residuals and the number of experiments in Figure 14b, the distribution of the residuals is uniform, which indicates that the residuals are small and have little effect on the experimental results. From the relationship between the measured and predicted values in Figure 14c, the experimental data points are close to the fitted line, which indicates that the error between the experimental and predicted values is small.

Table 5 presents the results of the response surface ANOVA. The regression model *p*-value < 0.0001 indicates that the model is highly significant; at *p* > 0.1, the model is not significant [41]. As can be seen from Table 5, factors A, B, C, AB, BC, A^2^, B^2^ and C^2^ are significant terms of the quadratic model. This indicates that H_2_O pressure, temperature and holding time have a more significant effect on the magnesium oxide yield, and that H_2_O pressure and temperature, and temperature and holding time have an interaction and squaring effect on the magnesium oxide yield. The F-value of the misfit term relative to the pure error is 1.29, which indicates that the misfit term is not significant. Due to noise, there is a 39.22% probability of having such a large F-value for the misfit term model, and the misfit term being insignificant is good for the fitted model. The regression coefficient of this quadratic model is 0.9995, the predicted R^2^ is 0.9961 and the adjusted R^2^ is 0.9990, and the difference between the two is 0.0029 (<0.2), which is a good degree of fit with a small test error. The following regression equation was obtained after fitting the model with the yield of MgO as the response value:

Y = 96.55 + 3.69A + 5.06B + 1.02C − 3.16AB + 0.65BC − 19.08A^2^ − 5.63B^2^ − 3.96C^2^ (12)

**Figure 15:**
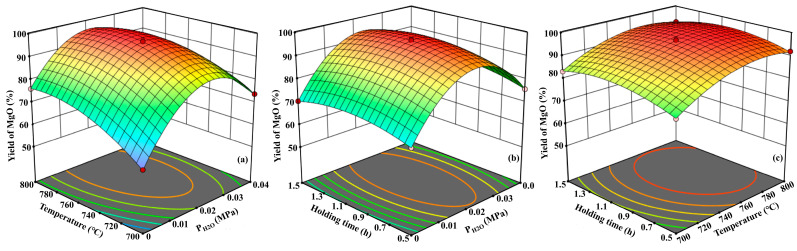
Interaction of various factors on MgO yield ((**a**) A + B; (**b**) A + C; (**c**) B + C).

The response surface analysis plots and corresponding contours of each parameter of the phosphate tailing pyrolysis process were derived from the regression equations, as shown in Figure 15. It can be seen that the greatest influence on the MgO yield is the temperature, followed by the H_2_O pressure and finally the holding time. The optimum pyrolysis conditions of phosphate tailings are as follows: A is 0.02 MPa, B is 758 °C and C is 1.08 h. Under this experimental condition, the MgO yield can be predicted to reach 97.4%.

The optimization results of the above pyrolysis conditions were experimentally verified, and the results obtained by repeating the experiment three times are shown in Table 6. The average yield of CaO and MgO in the three parallel experiments was 98.1% and 97.3%, respectively, and the average activity of MgO was 31.6, which is basically consistent with the predicted results, indicating that the above regression model has good accuracy and reliability.

### 3.4. Existence and Distribution of Calcium and Magnesium in Pyrolysis Phosphate Tailings

#### 3.4.1. Mineral Phase Composition

**Figure 16:**
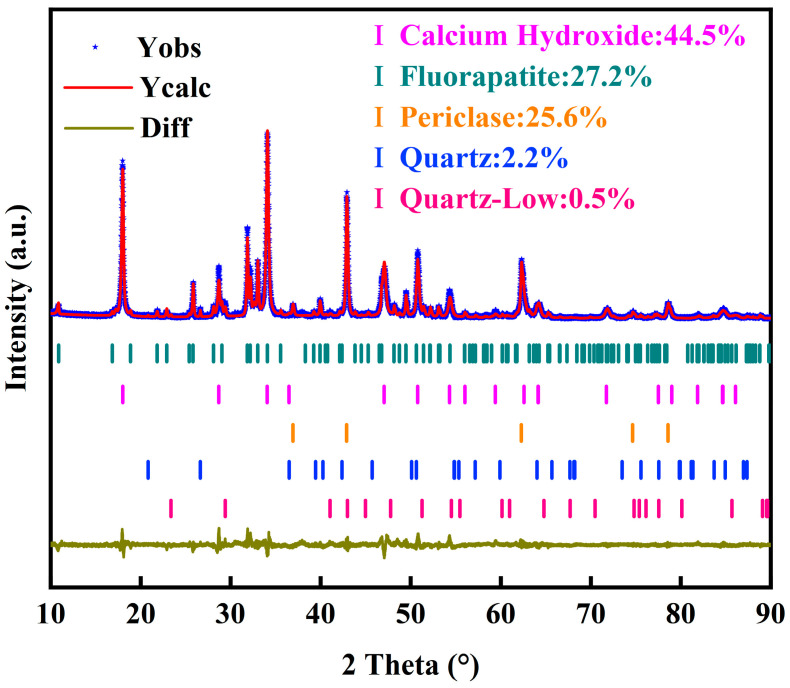
Rietveld refinement of pyrolyzed phosphate tailings under optimal pyrolysis conditions.

The XRD pattern of pyrolysis phosphate tailings was simulated by the Rietveld method and its physical phase composition was quantitatively analyzed, and the results are shown in Figure 16. As can be seen in Figure 16, the main mineral phase compositions in the pyrolyzed phosphate tailings were calcium hydroxide, magnesium oxide, fluorapatite and quartz, of which the calcium hydroxide content was the highest, at 44.5%, followed by a fluorapatite content of 27.2% and a magnesium oxide content of 25.6%, and the quartz content was the lowest, at 2.2%; the results of the crystal structure of each physical phase fitting are shown in Table 7.

**Figure 17:**
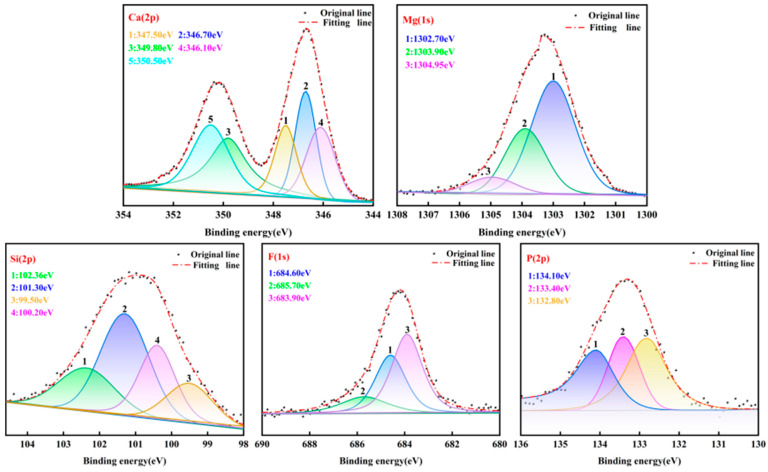
High-resolution XPS spectra of Ca2p, Mg1s, Si2p, F1s and P2p in pyrolytic phosphate tailings under optimal pyrolysis conditions.

The XPS spectra of Ca, Mg, Si, F and P are shown in Figure 17. The X-ray photoelectron spectroscopy database of the National Institute of Standards and Technology (NIST) of the United States was used in this work [42]. In addition to the main phases Ca(OH)_2_, MgO, Ca_5_(PO_4_)_3_F and SiO_2_ in the pyrolyzed phosphate tailings, the presence of CaSiO_3_ was found at a Si2p spectral binding energy of 102.36 eV, which suggests that Ca(OH)_2_ in the pyrolysis product does react with SiO_2_ to form CaSiO_3_ (no MgSiO_3_ was found). This result is consistent with the thermodynamic computational simulations. Moreover, the F1s spectral binding energies were 685.70 eV, 684.80 eV and 683.90 eV, which represent the presence of CaF_2_, MgF_2_ and KF, respectively. P2p spectral splitting fitted three peaks corresponding to binding energies of 134.10 eV, 133.40 eV and 132.80 eV, which were analyzed to be derived from NaH_2_PO_4_, CaHPO_4_ and Na_2_HPO_4_, respectively. It can be seen that there are also very small amounts of other impurity components in the pyrolyzed phosphate tailings.

#### 3.4.2. Microscopic Morphology

**Figure 18:**
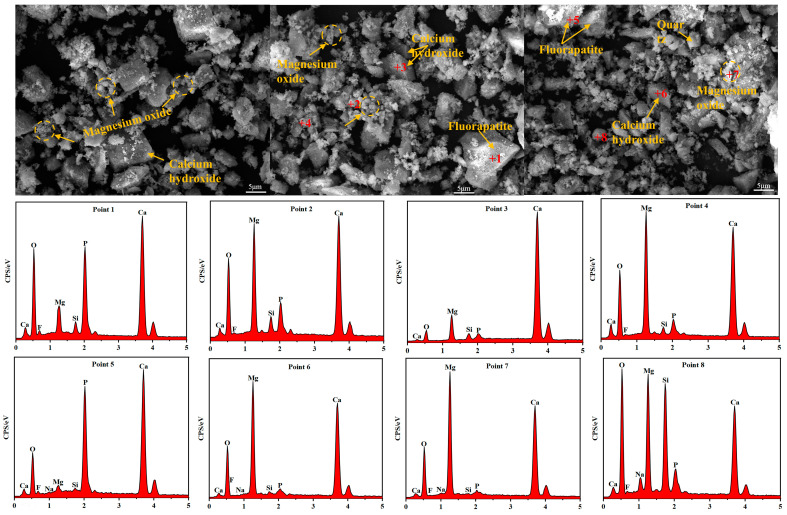
Microstructure of pyrolyzed phosphate tailings.

The microscopic morphology of the pyrolyzed phosphate tailings is shown in Figure 18. As seen in Figure 18, the pyrolysis products have diverse morphologies, with obviously flocculent, smooth and large agglomerates, being similar to cubic lumps and small spherical materials. According to the analysis of the EDS point scan results, it can be seen that the small spherical agglomerates at point 1 and the loose agglomerates at point 5 are mainly fluorapatite [43], the rhombic lumps at point 3 and point 6 mainly represent calcium hydroxide [44,45], and the crumbly material at point 4 and flocculent material at point 7 are mainly magnesium oxide [46]. It can be seen from the EDS results of the irregular large lumps at point 2, the rhombic lumps at point 6 and the flocculated material at point 7 that cubic calcium hydroxide has a large amount of magnesium oxide attached to its surface, while the 8-point spherical material is a silica-containing material, which is mainly calcium silicate or silicon dioxide, and combined with the results of the correlation scatter plot of Mg-Si, the presence of magnesium silicate is considered very unlikely.

#### 3.4.3. Distribution of Calcium and Magnesium in Pyrolytic Phosphate Tailings

**Figure 19:**
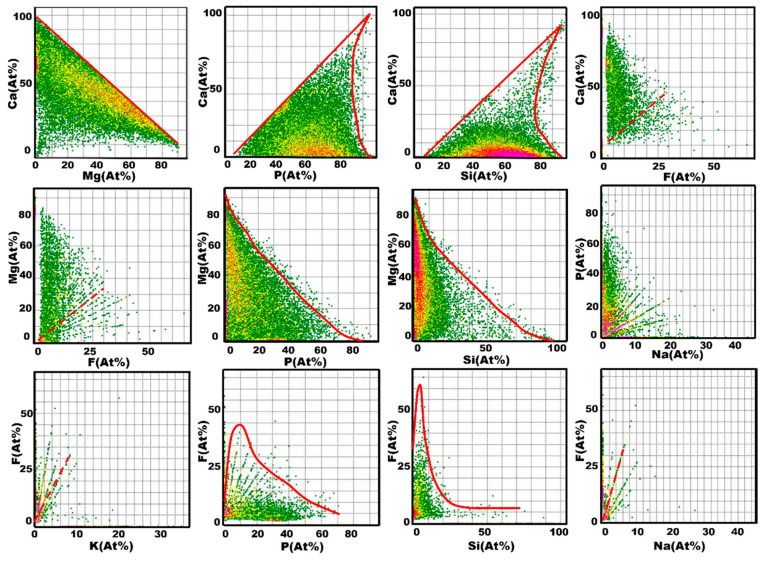
Quantitative EPMA scatter plot data for Ca, Mg, Si, F, P, K and Na (at.%).

The correlation scatter plot of the content of each element in the pyrolyzed phosphate tailings is shown in Figure 19. From the results of the correlation scatter plots of Ca-Mg, Ca-P, Ca-Si, Ca-F and P-F, it can be seen that the content of Ca had an obvious negative correlation with the content of Mg, indicating that Ca and Mg in the calcined phosphate tailings were no longer forming correlation compounds, and that the calcium magnesium carbonates had been decomposed completely. The content of Ca was positively correlated with the content of P and Si up to 80%, and the positive correlation was significantly weakened after exceeding this percentage. The content of Ca was significantly positively correlated with the content of F up to 25%, and the correlation was weakened after exceeding this value, and the content of F and the content of P also showed this trend. These results indicate the presence of fluorapatite, calcium fluoride, calcium hydrogen phosphate and calcium silicate in the pyrolysis products.

The correlation scatter plot results of Mg-Si, Mg-P and Mg-F show that the content of Mg had a negative correlation with the content of Si, and the negative correlation with the content of P was more obvious. The content of Mg had a weak positive correlation with the content of F, which indicates that there was a very small possibility of the existence of magnesium silicate and magnesium phosphate in the pyrolyzed phosphate tailings. Combined with the previous XPS results, it can be seen that there was also magnesium fluoride in the sample, and the scatter plot results are consistent with the previous results.

The content of P showed a certain positive correlation with the content of Na, and combined with the results of XPS analysis, it was concluded that there was also sodium hydrogen sulphate in the product.

The correlation scatter plots of F-K, F-Si, and F-Na suggest that the content of F showed a weak positive correlation with the content of K and Na. The scatter distribution shows that the contents of K and Na were small and therefore could not be detected in the previous XRD characterization method. The content of F showed a weak positive correlation with the content of Si in a very low range, indicating that a small amount of Si and F formed the corresponding compounds, which might be caused by the presence of K_2_SiF_6_ [44].

**Figure 20:**
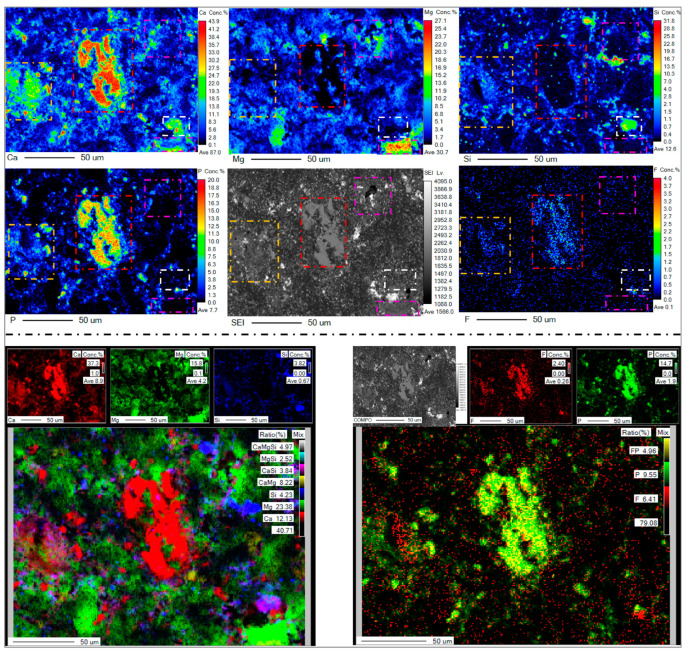
EPMA-mapped images of distribution of Ca, Mg, Si, F and P.

The EPMA surface scanning mapping results of Ca, Mg and other elements in the pyrolyzed phosphate tailings are shown in Figure 20. It can be seen from Figure 20 that the distribution of Ca and Mg elements was uneven, and the overall distribution contained small particles or was even powder-intensive. By carefully comparing the distribution maps of each element, it can be seen that the content of the Ca element was also relatively high in the area with a high content of P and F elements, and the content of Mg and Si was relatively low (such as the red box area in the picture). In the area with a high content of the P element, the content of Ca was obviously higher, and the content of F and Mg was relatively small (such as the orange box area in the figure). The content of Ca was significantly higher in the area with a higher Si content, and the content of Mg, P and F was lower (as shown in the white box area in the figure). The content of other elements in the area with a high content of Ca and Mg was obviously low (such as the purple box area in the figure). These results indicate that the distribution of P and F in pyrolyzed phosphate tailings had a high correlation with the distribution of Ca, the distribution of P had a high correlation with the distribution of Ca, and the distribution of Si in pyrolyzed phosphate tailings had a high correlation with the distribution of Ca. These results are in agreement with the quantitative EPMA analysis, i.e., Ca and Mg no longer form associative compounds, with Ca mainly in the form of Ca_5_(PO_4_)_3_F, CaHPO_4_, Ca(OH)_2_, CaF_2_ and CaSiO_3_, and Mg mainly as MgO, Mg(OH)_2_ and MgF_2_, which opens up the possibility of further separation of calcium and magnesium.

## 4. Conclusions

(1) Compared with N_2_, air and CO_2_ atmospheres, the pyrolysis process of phosphate tailings under a H_2_O atmosphere was changed from two stages to one stage, which was mainly the decomposition of CaMg(CO_3_)_2_ under the action of water molecules to generate Ca(OH)_2_ and Mg(OH)_2_ and to release CO_2,_ as well as the decomposition of the intermediate products Ca(OH)_2_ and Mg(OH)_2_. The starting decomposition temperature of this process was lowered to 500 °C, and the decomposition time was shortened to 30 min, with the pyrolysis rate being faster, which indicates that the decomposition of phosphate tailings by water vapor had an obvious promotion effect.

(2) Under the H_2_O atmosphere, the influence of each factor on the pyrolysis process of phosphate tailings was in the following order: temperature > H_2_O pressure > holding time. When the H_2_O pressure was 0.02 MPa, the temperature was 758 °C, the holding time was 1.08 h and the heating rate was 10 K/min, the MgO and CaO yield was the highest, at 97.3% and 98.1%, respectively, and the MgO activity was 31.6%. The CaMg(CO_3_)_2_ decomposition of the phosphate tailings was more complete.

(3) After the pyrolysis of phosphate tailings in the H_2_O atmosphere, its main component was Ca(OH)_2_ at 44.5%, followed by Ca_5_(PO_4_)_3_F at 27.2%, MgO at 25.6%, and SiO_2_ with the lowest content of 2.2%. The distribution of Ca and Mg elements in the phosphate tailings after pyrolysis showed a negative correlation, indicating that the two no longer formed associated compounds, in which Ca mainly existed in the form of Ca_5_(PO_4_)_3_F, CaHPO_4_, Ca(OH)_2_, CaF_2_ and CaSiO_3_, and Mg mainly existed in the form of MgO, Mg(OH)_2_ and MgF_2_, which provides a possibility for further separation of calcium and magnesium.

## Figures and Tables

**Table 1:** Chemical composition of phosphate tailings (mass%).

CaO	MgO	P_2_O_5_	SiO_2_	Fe_2_O_3_	Al_2_O_3_	F	SO_3_	Na_2_O	K_2_O	CO_2_	Others
33.96	18.49	5.58	1.97	0.26	0.29	0.61	0.70	0.04	0.01	38.03	0.06

**Table 2:** The possible reaction equations of thermal decomposition of the phosphate tailings in a H_2_O atmosphere.

Reaction	Process
R_1_	CaMg(CO_3_)_2_ + 2H_2_O = Ca(OH)_2_ + Mg(OH)_2_ + 2CO_2_(g)
R_2_	CaMg(CO_3_)_2_ + H_2_O = Ca(OH)_2_ + MgO + 2CO_2_(g)
R_3_	CaMg(CO_3_)_2_ + H_2_O = CaO + Mg(OH)_2_ + 2CO_2_(g)
R_4_	Mg(OH)_2_ = MgO + H_2_O(g)
R_5_	Ca(OH)_2_ = CaO + H_2_O(g)
R_6_	Mg(OH)_2_ + SiO_2_ = MgSiO_3_ + H_2_O(g)
R_7_	Ca(OH)_2_ + SiO_2_ = CaSiO_3_ + H_2_O(g)
R_8_	MgO + SiO_2_ = MgSiO_3_
R_9_	CaO + SiO_2_ = CaSiO_3_
R_10_	2Ca_5_(PO_4_)_3_F + 2H_2_O = Ca(OH)_2_ + 2HF(g) + 3Ca_3_(PO_4_)_2_

**Table 3:** Factors and levels designed with Box–Behnken.

Variable		Symbol Coded Level		
	Uncoded Coded		Low Center High	
		−1	0	1
H_2_O pressure	A	0.00	0.02	0.04
Temperature	B	700	750	800
Holding time	C	0.5	1	1.5

**Table 4:** Box–Behnken experimental results.

Std No.	Coded Level of Variables			Actual Level of Variables			Yield of MgO (%)
	A	B	C	A	B	C	
(1)	0	0	0	0.02	750	1	96.6
(2)	1	0	−1	0.04	750	0.5	75.8
(3)	0	1	1	0.02	800	1.5	92.6
(4)	−1	−1	0	0.00	700	1	60.2
(5)	1	−1	0	0.04	700	1	73.9
(6)	0	0	0	0.02	750	1	96.9
(7)	0	−1	−1	0.02	700	0.5	80.1
(8)	0	0	0	0.02	750	1	96.6
(9)	−1	1	0	0.00	800	1	76.1
(10)	−1	0	−1	0.00	750	0.5	69.1
(11)	0	0	0	0.02	750	1	95.9
(12)	−1	0	1	0.00	750	1.5	70.6
(13)	1	1	0	0.04	800	1	77.2
(14)	0	−1	1	0.02	700	1.5	83.3
(15)	0	0	0	0.02	750	1	96.6
(16)	0	1	−1	0.02	800	0.5	91.9
(17)	1	0	1	0.04	750	1.5	78.7

**Table 5:** Analysis of variance of MgO yield response surface.

Source	Sum of Squares	df	Mean Square	F-Value	*p*-Value	
Model	2203.54	9	244.84	1709.15	<0.0001	significant
A	109.11	1	109.11	761.66	<0.0001	significant
B	204.47	1	204.47	1427.34	<0.0001	significant
C	8.25	1	8.25	57.57	0.0001	significant
AB	40.04	1	40.04	279.51	<0.0001	significant
AC	0.4153	1	0.4153	2.90	0.1324	
BC	1.68	1	1.68	11.70	0.0111	significant
A^2^	1533.08	1	1533.08	10702.03	<0.0001	significant
B^2^	133.67	1	133.67	933.09	<0.0001	significant
C^2^	66.01	1	66.01	460.77	<0.0001	significant
Lack of Fit	0.4931	3	0.1644	1.29	0.3299	not significant
Pure Error	0.5096	4	0.1274			
R^2^	0.9995					
Adjusted R^2^	0.9990					
Predicted R^2^	0.9961					

**Table 6:** Validation of optimization results of experiment.

	1	2	3	Average
CaO	98.4	97.8	98.2	98.1
MgO	97.2	97.6	97.3	97.3
Activity of MgO	31.9	31.4	31.6	31.6

**Table 7:** XRD fine-modified fitted crystal structure data.

Mineral Phase	Lattices Parameters						Space Group	Volume (Å)
	a (Å)	b (Å)	c (Å)	α (°)	β (°)	γ (°)		
Ca_5_(PO_4_)_3_F	9.39121(15)	9.39121(15)	6.89328(16)	90	90	120	P63/m	526.502(21)
Ca(OH)_2_	3.59186(7)	3.59185(7)	4.9213(2)	90	90	120	P-3m1	54.985(3)
MgO	4.21322(5)	4.21322(5)	4.21322(5)	90	90	90	Fm-3m	74.790(3)
SiO_2_	4.39251(90)	4.39251(90)	5.0433(22)	90	90	120	P3221	84.269(50)
SiO_2_-low	4.9195(14)	4.9195(14)	5.4032(26)	90	90	120	P3121	113.247(86)

## Data Availability

Data are contained within the article.
